# Can massage therapy help cardiac patients? A literature review plus a case study

**DOI:** 10.1186/cc12434

**Published:** 2013-03-19

**Authors:** A Fenlon

**Affiliations:** 1 St Georges NHS Trust, London, UK

## Introduction

A literature review was performed to assess whether massage benefits patients postoperatively following coronary bypass grafts (CABG) and or valve replacement/repair. A case study on a patient who had suffered a hypoxic brain post cardiac arrest was conducted.

## Methods

A review on MEDLINE and Cochrane using search terms massage, cardiac and ICU identified nine research papers on the benefits of massage postoperatively for the aforementioned patient group. Other papers were listed but unrelated to cardiac surgery. None of the nine papers identified for this review were ICU specific in the title but the ICU was mentioned in the main text body. For the purpose of this review the selected papers are researching the effects of massage on physiological parameters, anxiety, pain, calm and perceived stress indicators in the CABG and/or valve repair/replacement. Out of these nine papers, one is British (2002). Five are American (2006 to 2012), two are Brazilian (2010) and one is an Indian paper (2010). All papers are randomised control trials (RCTs). Papers written prior to 1999 were excluded from this literature review.

## Results

Research from 1999 states there are methodological errors in prior papers and very few large-scale studies prior to this date, destabilising the validity and reliability of research from the papers written before 1999. The later research suggests that any change in the measured physiological parameters of blood pressure, heart rate and respiratory rate are insignificant (*P >*0.05). Pain, anxiety, rest and a calm score perform better with *P >*4.71 overall. Another American study showed that the length of stay is reduced if a patient receives healing touch postoperatively. However, in one RCT pain and anxiety increases and in some case SpO_2 _decreases. A case study was chosen by the author and the results support the benefits of massage with the cardiac patient group.

## Conclusion

There appears to be some benefits but larger-scale studies are required within this and other ICUs in Britain. The author will be inquiring at her workplace whether further studies can be performed and whether she can initiate the research. She will identify further case studies and trial massage on select patients. The author recommends training for nurses interested in massage therapy so a bigger caseload can be identified.

